# 2-(3-Oxo-3,4-dihydro-2*H*-1,4-benzo­thia­zin-4-yl)acetic acid monohydrate

**DOI:** 10.1107/S1600536809034977

**Published:** 2009-09-05

**Authors:** Hoong-Kun Fun, Wan-Sin Loh, G. Janardhana, A. M. A. Khader, B. Kalluraya

**Affiliations:** a X-ray Crystallography Unit, School of Physics, Universiti Sains Malaysia, 11800 USM, Penang, Malaysia; b Department of Studies in Chemistry, Mangalore University, Mangalagangothri, Mangalore 574 199, India

## Abstract

In the title compound, C_10_H_9_NO_3_S·H_2_O, the thio­morpholine ring exists in a conformation inter­mediate between twist-boat and half-chair. An inter­molecular O—H⋯O hydrogen bond links the acid and water mol­ecules together. In the crystal packing, inter­molecular O—H⋯O and C—H⋯O hydrogen bonds link the mol­ecules into a three-dimensional network.

## Related literature

For the biological activity of 4*H*-benzo(1,4)thia­zine, see: Armenise *et al.* (1991 ▶); Gupta *et al.* (1993 ▶); Fringuelli *et al.* (2005 ▶). For medical applications of sulfone derivatives of 4*H*-benzo(1,4)thia­zine, see: Shinji & Koshiro (1995 ▶); Szule *et al.* (1988 ▶); Culbertson (1991 ▶). For a related structure, see: Zhang *et al.* (2008 ▶). For bond-length data, see: Allen *et al.* (1987 ▶). For ring puckering parameters, see: Cremer & Pople (1975 ▶). For the stability of the temperature controller used for the data collection, see: Cosier & Glazer (1986 ▶).
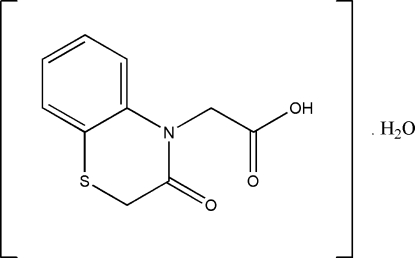


## Experimental

### 

#### Crystal data

C_10_H_9_NO_3_S·H_2_O
*M* *_r_* = 241.26
Monoclinic, 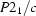

*a* = 7.5897 (1) Å
*b* = 9.2208 (2) Å
*c* = 15.6701 (3) Å
β = 94.336 (1)°
*V* = 1093.50 (3) Å^3^
*Z* = 4
Mo *K*α radiation
μ = 0.29 mm^−1^
*T* = 100 K
0.49 × 0.34 × 0.11 mm

#### Data collection

Bruker SMART APEXII CCD area-detector diffractometer
Absorption correction: multi-scan (**SADABS**; Bruker, 2005 ▶) *T*_min_ = 0.870, *T*_max_ = 0.969
25955 measured reflections
4859 independent reflections
3833 reflections with *I* > 2σ(*I*)
*R*_int_ = 0.036

#### Refinement

*R*[*F* ^2^ > 2σ(*F* ^2^)] = 0.040
*wR*(*F* ^2^) = 0.128
*S* = 0.83
4859 reflections
157 parameters
H atoms treated by a mixture of independent and constrained refinement
Δρ_max_ = 0.54 e Å^−3^
Δρ_min_ = −0.26 e Å^−3^

### 

Data collection: *APEX2* (Bruker, 2005 ▶); cell refinement: *SAINT* (Bruker, 2005 ▶); data reduction: *SAINT*; program(s) used to solve structure: *SHELXTL* (Sheldrick, 2008 ▶); program(s) used to refine structure: *SHELXTL*; molecular graphics: *SHELXTL*; software used to prepare material for publication: *SHELXTL* and *PLATON* (Spek, 2009 ▶).

## Supplementary Material

Crystal structure: contains datablocks global, I. DOI: 10.1107/S1600536809034977/sj2641sup1.cif

Structure factors: contains datablocks I. DOI: 10.1107/S1600536809034977/sj2641Isup2.hkl

Additional supplementary materials:  crystallographic information; 3D view; checkCIF report

## Figures and Tables

**Table 1:** Hydrogen-bond geometry (Å, °)

*D*—H⋯*A*	*D*—H	H⋯*A*	*D*⋯*A*	*D*—H⋯*A*
O2—H1*O*2⋯O1*W*^i^	0.93 (2)	1.62 (2)	2.5384 (13)	168 (3)
O1*W*—H2*W*1⋯O3^ii^	0.85 (2)	1.96 (2)	2.7893 (13)	168 (2)
O1*W*—H1*W*1⋯O1	0.90 (2)	1.85 (2)	2.7221 (13)	163.4 (19)
C2—H2*A*⋯O1*W*^iii^	0.93	2.51	3.3666 (15)	153
C9—H9*A*⋯O2^iv^	0.97	2.58	3.4429 (14)	149

Symmetry codes: (i) 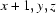; (ii) 
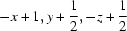
; (iii) 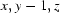; (iv) 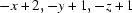.

**Fig. 1.**
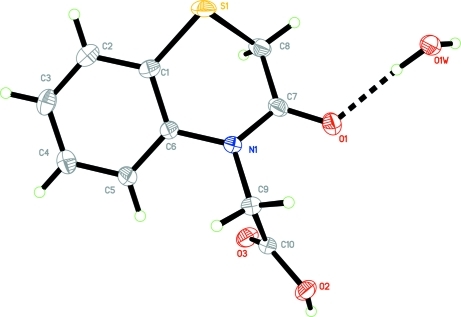
The molecular structure of the title compound, showing 50% probability displacement ellipsoids and the atom numbering scheme. The hydrogen bond is drawn as a dashed line.

**Fig. 2.**
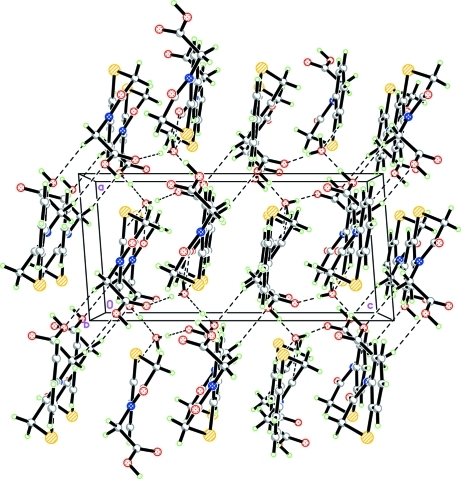
The crystal packing of the title compound, viewed along b axis. Intermolecular hydrogen bonds are shown by dashed lines.

## supplementary crystallographic information

### Comment

A number of molecules containing the 4*H*-benzo(1,4)thiazine nucleus in their structures exhibit a broad spectrum of biological activity, including antibacterial (Armenise *et al.*, 1991), anticancer (Gupta *et al.*, 1993), anti-rheumatic, anti-allergic, vasorelaxant, anti-arrhythmic and anti-hypertensive (Fringuelli *et al.*, 2005) properties. The sulfone derivatives of 4*H*-benzo(1,4)thiazine have been reported to find a number of applications in medicine (Shinji & Koshiro, 1995; Szule *et al.*, 1988; Culbertson, 1991). On the basis of these considerations, our particular attention was paid to the preparation of derivatives of (3-*oxo*-3,4-dihydro-2*H*-1,4-benzothiazin-4-yl)acetic acid and we report here the structure of the title 4-benzothiazine derivative.

The asymmetric unit of the title compound (Fig. 1), contains one (3-*oxo*-3,4-dihydro-2*H*-1,4-benzothiazin-4-yl)acetic acid and one water molecule. The bond lengths (Allen *et al.*, 1987) and angles in the molecule are within normal ranges. The thiomorpholine ring (C1/C6–C8/N1/S1) exists in a conformation intermediate between twist-boat and half-chair and it is comparable to a closely related structure (Zhang *et al.*, 2008). The puckering parameters (Cremer & Pople, 1975) are Q = 0.6852 (9) Å; Θ = 112.69 (8)° and φ = 152.79 (10)°. An intermolecular O1W1—H1W1···O1 hydrogen bond links the acid and water molecules together. In the crystal packing (Fig. 2), intermolecular O2—H1O2···O1W, O1W—H2W1···O3, C2—H2A···O1W and C9—H10A···O2 hydrogen bonds (Table 1) link the molecules into three-dimensional network.

### Experimental

A solution of potassium hydroxide (5.85 mmol) in water (10 ml) was added to the solution of ethyl (3-*oxo*-3,4-dihydro-2*H*-1,4-benzothiazin-4-yl)acetate (3.9 mmol) in ethanol (10 ml). The resulting reaction mixture was stirred at room temperature for 24 h and the reaction completion was checked by TLC. The reaction mixture was poured into water and acidified with 4 M HCl to form (3-*oxo*-3,4-dihydro-2*H*-1,4-benzothiazin-4-yl)acetic acid as colourless solid. Single crystals suitable for X-ray analysis were obtained by crystallization from dichloromethane under slow evaporation (*M.p.* 338 K).

### Refinement

Atom H1O2, H1W1 and H2W1 were located in a difference map and were refined freely. Other H atoms were positioned geometrically [C—H = 0.93 or 0.97 Å] and were refined using a riding model, with *U*_iso_(H) = 1.2 *U*_eq_(C).

### Crystal data

**Table:**

C_10_H_9_NO_3_S·H_2_O	*F*(000) = 504
*M**_r_* = 241.26	*D*_x_ = 1.465 Mg m^−^^3^
Monoclinic, *P*2_1_/*c*	Mo *K*α radiation, λ = 0.71073 Å
Hall symbol: -P 2ybc	Cell parameters from 7672 reflections
*a* = 7.5897 (1) Å	θ = 3.4–33.1°
*b* = 9.2208 (2) Å	µ = 0.29 mm^−^^1^
*c* = 15.6701 (3) Å	*T* = 100 K
β = 94.336 (1)°	Block, colourless
*V* = 1093.50 (3) Å^3^	0.49 × 0.34 × 0.11 mm
*Z* = 4	

### Data collection

**Table:**

Bruker SMART APEXII CCD area-detector diffractometer	4859 independent reflections
Radiation source: fine-focus sealed tube	3833 reflections with *I* > 2σ(*I*)
graphite	*R*_int_ = 0.036
φ and ω scans	θ_max_ = 35.1°, θ_min_ = 2.6°
Absorption correction: multi-scan (*SADABS*; Bruker, 2005)	*h* = −12→12
*T*_min_ = 0.870, *T*_max_ = 0.969	*k* = −14→13
25955 measured reflections	*l* = −23→25

### Refinement

**Table:**

Refinement on *F*^2^	Primary atom site location: structure-invariant direct methods
Least-squares matrix: full	Secondary atom site location: difference Fourier map
*R*[*F*^2^ > 2σ(*F*^2^)] = 0.040	Hydrogen site location: inferred from neighbouring sites
*wR*(*F*^2^) = 0.128	H atoms treated by a mixture of independent and constrained refinement
*S* = 0.83	*w* = 1/[σ^2^(*F*_o_^2^) + (0.0915*P*)^2^ + 0.3956*P*] where *P* = (*F*_o_^2^ + 2*F*_c_^2^)/3
4859 reflections	(Δ/σ)_max_ = 0.001?
157 parameters	Δρ_max_ = 0.54 e Å^−^^3^
0 restraints	Δρ_min_ = −0.26 e Å^−^^3^

### Special details

**Table:**

Experimental. The crystal was placed in the cold stream of an Oxford Cyrosystems Cobra open-flow nitrogen cryostat (Cosier & Glazer, 1986) operating at 100.0 (1) K.
Geometry. All e.s.d.'s (except the e.s.d. in the dihedral angle between two l.s. planes) are estimated using the full covariance matrix. The cell e.s.d.'s are taken into account individually in the estimation of e.s.d.'s in distances, angles and torsion angles; correlations between e.s.d.'s in cell parameters are only used when they are defined by crystal symmetry. An approximate (isotropic) treatment of cell e.s.d.'s is used for estimating e.s.d.'s involving l.s. planes.
Refinement. Refinement of *F*^2^ against ALL reflections. The weighted *R*-factor *wR* and goodness of fit *S* are based on *F*^2^, conventional *R*-factors *R* are based on *F*, with *F* set to zero for negative *F*^2^. The threshold expression of *F*^2^ > σ(*F*^2^) is used only for calculating *R*-factors(gt) *etc*. and is not relevant to the choice of reflections for refinement. *R*-factors based on *F*^2^ are statistically about twice as large as those based on *F*, and *R*- factors based on ALL data will be even larger.

### Fractional atomic coordinates and isotropic or equivalent isotropic displacement parameters (Å^2^)

**Table:**

	*x*	*y*	*z*	*U*_iso_*/*U*_eq_	
S1	0.25290 (3)	0.20298 (3)	0.368735 (18)	0.02391 (8)	
O1W	0.17478 (12)	0.72939 (10)	0.31220 (6)	0.02515 (17)	
O1	0.47141 (12)	0.56427 (9)	0.33767 (6)	0.02699 (18)	
O2	0.94362 (11)	0.62615 (9)	0.40417 (6)	0.02360 (16)	
O3	0.90204 (12)	0.44113 (9)	0.31220 (6)	0.02548 (17)	
C1	0.46033 (14)	0.11958 (11)	0.38793 (6)	0.01900 (18)	
C2	0.47444 (16)	−0.03074 (12)	0.39590 (7)	0.0233 (2)	
H2A	0.3733	−0.0879	0.3900	0.028*	
C3	0.63845 (17)	−0.09543 (12)	0.41263 (7)	0.0247 (2)	
H3A	0.6469	−0.1955	0.4193	0.030*	
C4	0.78984 (16)	−0.01067 (12)	0.41946 (7)	0.0246 (2)	
H4A	0.8999	−0.0544	0.4296	0.030*	
C5	0.77815 (14)	0.13934 (12)	0.41127 (7)	0.02197 (19)	
H5A	0.8801	0.1956	0.4156	0.026*	
C6	0.61270 (13)	0.20523 (11)	0.39649 (6)	0.01770 (17)	
N1	0.59863 (11)	0.35987 (9)	0.39215 (6)	0.01904 (16)	
C7	0.46962 (14)	0.43038 (12)	0.34290 (7)	0.02067 (19)	
C8	0.32911 (14)	0.33868 (13)	0.29697 (7)	0.0234 (2)	
H8A	0.3764	0.2918	0.2482	0.028*	
H8B	0.2309	0.3995	0.2760	0.028*	
C9	0.73286 (14)	0.45083 (11)	0.43631 (7)	0.02047 (18)	
H9A	0.7934	0.3959	0.4825	0.025*	
H9B	0.6764	0.5333	0.4613	0.025*	
C10	0.86713 (13)	0.50482 (11)	0.37642 (7)	0.01941 (18)	
H1O2	1.028 (3)	0.652 (3)	0.3672 (15)	0.064 (7)*	
H2W1	0.155 (3)	0.784 (2)	0.2691 (15)	0.056 (6)*	
H1W1	0.270 (3)	0.672 (2)	0.3095 (13)	0.043 (5)*	

### Atomic displacement parameters (Å^2^)

**Table:**

	*U*^11^	*U*^22^	*U*^33^	*U*^12^	*U*^13^	*U*^23^
S1	0.01398 (12)	0.03081 (15)	0.02713 (14)	−0.00430 (9)	0.00291 (9)	0.00246 (9)
O1W	0.0185 (4)	0.0229 (4)	0.0340 (4)	0.0004 (3)	0.0017 (3)	0.0072 (3)
O1	0.0224 (4)	0.0214 (4)	0.0371 (5)	0.0033 (3)	0.0020 (3)	0.0071 (3)
O2	0.0222 (4)	0.0184 (3)	0.0304 (4)	−0.0051 (3)	0.0030 (3)	−0.0026 (3)
O3	0.0231 (4)	0.0232 (4)	0.0306 (4)	−0.0046 (3)	0.0049 (3)	−0.0046 (3)
C1	0.0179 (4)	0.0209 (4)	0.0184 (4)	−0.0039 (3)	0.0028 (3)	−0.0002 (3)
C2	0.0279 (5)	0.0217 (5)	0.0208 (4)	−0.0064 (4)	0.0046 (4)	−0.0016 (3)
C3	0.0358 (6)	0.0175 (4)	0.0211 (4)	−0.0004 (4)	0.0050 (4)	−0.0006 (3)
C4	0.0264 (5)	0.0226 (5)	0.0251 (5)	0.0052 (4)	0.0030 (4)	0.0020 (4)
C5	0.0169 (4)	0.0206 (4)	0.0284 (5)	0.0016 (3)	0.0019 (3)	0.0028 (4)
C6	0.0162 (4)	0.0169 (4)	0.0201 (4)	−0.0009 (3)	0.0022 (3)	0.0013 (3)
N1	0.0136 (3)	0.0176 (4)	0.0256 (4)	−0.0004 (3)	−0.0006 (3)	0.0029 (3)
C7	0.0150 (4)	0.0233 (5)	0.0239 (4)	0.0020 (3)	0.0027 (3)	0.0048 (3)
C8	0.0169 (4)	0.0296 (5)	0.0233 (5)	−0.0003 (4)	−0.0008 (3)	0.0047 (4)
C9	0.0172 (4)	0.0196 (4)	0.0243 (4)	−0.0020 (3)	−0.0001 (3)	0.0002 (3)
C10	0.0147 (4)	0.0169 (4)	0.0262 (5)	0.0000 (3)	−0.0009 (3)	0.0000 (3)

### Geometric parameters (Å, °)

**Table:**

S1—C1	1.7575 (11)	C4—C5	1.3914 (16)
S1—C8	1.8064 (12)	C4—H4A	0.9300
O1W—H2W1	0.85 (2)	C5—C6	1.3984 (15)
O1W—H1W1	0.90 (2)	C5—H5A	0.9300
O1—C7	1.2374 (13)	C6—N1	1.4311 (13)
O2—C10	1.3189 (13)	N1—C7	1.3648 (13)
O2—H1O2	0.93 (3)	N1—C9	1.4539 (13)
O3—C10	1.2116 (14)	C7—C8	1.5014 (16)
C1—C2	1.3951 (15)	C8—H8A	0.9700
C1—C6	1.3985 (14)	C8—H8B	0.9700
C2—C3	1.3873 (17)	C9—C10	1.5205 (16)
C2—H2A	0.9300	C9—H9A	0.9700
C3—C4	1.3871 (17)	C9—H9B	0.9700
C3—H3A	0.9300		
			
C1—S1—C8	94.86 (5)	C7—N1—C6	123.32 (9)
H2W1—O1W—H1W1	114 (2)	C7—N1—C9	116.19 (8)
C10—O2—H1O2	108.8 (14)	C6—N1—C9	120.33 (8)
C2—C1—C6	119.68 (10)	O1—C7—N1	120.11 (10)
C2—C1—S1	120.78 (8)	O1—C7—C8	122.76 (10)
C6—C1—S1	119.53 (8)	N1—C7—C8	117.12 (9)
C3—C2—C1	120.38 (10)	C7—C8—S1	109.94 (7)
C3—C2—H2A	119.8	C7—C8—H8A	109.7
C1—C2—H2A	119.8	S1—C8—H8A	109.7
C4—C3—C2	119.90 (10)	C7—C8—H8B	109.7
C4—C3—H3A	120.0	S1—C8—H8B	109.7
C2—C3—H3A	120.0	H8A—C8—H8B	108.2
C3—C4—C5	120.42 (11)	N1—C9—C10	111.92 (9)
C3—C4—H4A	119.8	N1—C9—H9A	109.2
C5—C4—H4A	119.8	C10—C9—H9A	109.2
C4—C5—C6	119.83 (10)	N1—C9—H9B	109.2
C4—C5—H5A	120.1	C10—C9—H9B	109.2
C6—C5—H5A	120.1	H9A—C9—H9B	107.9
C1—C6—C5	119.75 (9)	O3—C10—O2	124.51 (10)
C1—C6—N1	119.99 (9)	O3—C10—C9	123.56 (9)
C5—C6—N1	120.24 (9)	O2—C10—C9	111.90 (9)
			
C8—S1—C1—C2	−142.00 (9)	C5—C6—N1—C7	149.31 (11)
C8—S1—C1—C6	38.91 (9)	C1—C6—N1—C9	152.60 (10)
C6—C1—C2—C3	0.30 (16)	C5—C6—N1—C9	−25.91 (14)
S1—C1—C2—C3	−178.80 (8)	C6—N1—C7—O1	−175.33 (10)
C1—C2—C3—C4	−1.55 (16)	C9—N1—C7—O1	0.07 (15)
C2—C3—C4—C5	1.24 (17)	C6—N1—C7—C8	4.85 (15)
C3—C4—C5—C6	0.33 (17)	C9—N1—C7—C8	−179.75 (9)
C2—C1—C6—C5	1.27 (15)	O1—C7—C8—S1	−134.43 (10)
S1—C1—C6—C5	−179.63 (8)	N1—C7—C8—S1	45.39 (12)
C2—C1—C6—N1	−177.25 (9)	C1—S1—C8—C7	−60.65 (8)
S1—C1—C6—N1	1.86 (13)	C7—N1—C9—C10	−77.23 (12)
C4—C5—C6—C1	−1.58 (16)	C6—N1—C9—C10	98.32 (11)
C4—C5—C6—N1	176.93 (10)	N1—C9—C10—O3	−26.48 (14)
C1—C6—N1—C7	−32.18 (15)	N1—C9—C10—O2	155.33 (9)

### Hydrogen-bond geometry (Å, °)

**Table:**

*D*—H···*A*	*D*—H	H···*A*	*D*···*A*	*D*—H···*A*
O2—H1O2···O1W^i^	0.93 (2)	1.62 (2)	2.5384 (13)	168 (3)
O1W—H2W1···O3^ii^	0.85 (2)	1.96 (2)	2.7893 (13)	168 (2)
O1W—H1W1···O1	0.90 (2)	1.85 (2)	2.7221 (13)	163.4 (19)
C2—H2A···O1W^iii^	0.93	2.51	3.3666 (15)	153
C9—H9A···O2^iv^	0.97	2.58	3.4429 (14)	149

Symmetry codes: (i) *x*+1, *y*, *z*; (ii) −*x*+1, *y*+1/2, −*z*+1/2; (iii) *x*, *y*−1, *z*; (iv) −*x*+2, −*y*+1, −*z*+1.
